# Methodology of the SORENTO clinical trial: a prospective, randomised, active-controlled phase 3 trial assessing the efficacy and safety of high exposure octreotide subcutaneous depot (CAM2029) in patients with GEP-NET

**DOI:** 10.1186/s13063-023-07834-8

**Published:** 2024-01-16

**Authors:** Simron Singh, Diego Ferone, Jaume Capdevila, Jennifer Ang Chan, Wouter W. de Herder, Daniel Halperin, Josh Mailman, Lisa Hellström, Hanna Liedman, Agneta Svedberg, Fredrik Tiberg

**Affiliations:** 1 Sunnybrook Health Sciences Center, Toronto, Canada; 2 Endocrinology, Department of Internal Medicine & Medical Specialties, University of Genova, Endocrinology Clinic IRCCS Ospedale Policlinico San Martino, Genova, Italy; 3 Vall d’Hebron University Hospital, Barcelona, Spain; 4 Dana-Farber Cancer Institute, Boston, MA USA; 5 Erasmus MC & Erasmus MC Cancer Institute, Rotterdam, The Netherlands; 6 The University of Texas MD Anderson Cancer Center, Houston, TX USA; 7 Northern California CarciNET Community, Oakland, CA USA; 8 Camurus AB, Lund, Sweden

**Keywords:** CAM2029, GEP-NET, High bioavailability, High plasma exposure, Octreotide, Randomised active-controlled, Somatostatin receptor ligands, SORENTO

## Abstract

**Background:**

The current standard of care (SoC) for the initial treatment of unresectable or metastatic well-differentiated gastroenteropancreatic neuroendocrine tumours (GEP-NET) requires initiation of first-generation somatostatin receptor ligand (SRL) therapy, octreotide and lanreotide, which provide safe and efficacious tumour/symptom control in most patients. However, disease progression can occur with SoC SRL treatment and the optimal dose response of SRL remains unknown. Octreotide subcutaneous depot (CAM2029) is a novel, long-acting, high-exposure formulation that has shown greater bioavailability and improved administration than octreotide long-acting release (LAR) with a well-tolerated safety profile. Retrospective data have highlighted a potential benefit of high-exposure SRL for improved disease control in patients who did not adequately respond to the current SoC SRL treatment. This trial will investigate the efficacy and tolerability of CAM2029 compared to the current SoC, including octreotide LAR and lanreotide autogel (ATG).

**Methods:**

SORENTO is a prospective, multicentre, randomised, active-controlled, open-label phase 3 trial aiming to demonstrate superiority of treatment with 20 mg octreotide subcutaneous depot (CAM2029) every 2 weeks (Q2W) compared to treatment with the Investigator’s choice of SRL therapy at standard doses for tumour control (octreotide LAR 30 mg or lanreotide ATG 120 mg every 4 weeks [Q4W]) as assessed by progression-free survival (PFS) in approximately 300 patients with unresectable/metastatic and well-differentiated GEP-NET. Upon confirmation of disease progression (determined by a Blinded Independent Review Committee [BIRC] and defined as per RECIST 1.1), patients may enter an open-label extension treatment period with once weekly dosing, to investigate the effects of higher frequency dosing. Overall survival follow-up will end a maximum of 2 years after primary analysis. The primary endpoint will be analysed after 194 confirmed PFS events.

**Discussion:**

This is the first trial investigating the efficacy of CAM2029 versus SoC SRL therapy using a head-to-head, superiority trial design. It is expected to be the first trial to investigate the efficacy of increased dosing frequency of a high-exposure SRL. A BIRC will limit bias and measurement variability and ensure high-quality efficacy data. Additionally, inclusion of patients with well-differentiated Grade 3 NET may elucidate treatment strategies for this rarely investigated patient population.

**Trial registration:**

ClinicalTrials.gov NCT05050942. Registered on 21st September 2021.

**Supplementary Information:**

The online version contains supplementary material available at 10.1186/s13063-023-07834-8.

## Administrative information

**Table:** 

Title	Methodology of the SORENTO clinical trial: a prospective, randomised, active-controlled phase 3 trial assessing the efficacy and safety of high exposure octreotide subcutaneous depot (CAM2029) in patients with GEP-NET
Trial registration	ClinicalTrials.gov: NCT05050942; EudraCT: 2021-000849-40
Protocol version	28^th^ November 2022 – Final Version 6
Funding	This clinical trial is funded by Camurus AB (Lund, Sweden)
Author details	^1^Simron Singh, MD; Sunnybrook Health Sciences Center, Toronto, Canada; Simron.Singh@sunnybrook.ca ^2^Diego Ferone, MD, PhD; Endocrinology, Department of Internal Medicine & Medical Specialties, University of Genova, Endocrinology Clinic IRCCS Ospedale Policlinico San Martino, Genova, Italy; ferone@unige.it ^3^Jaume Capdevila, MD, PhD; Vall d’Hebron University Hospital, Barcelona, Spain; jcapdevila@vhio.net ^4^Jennifer Ang Chan, MD; Dana-Farber Cancer Institute, Boston, MA, United States; jang@partners.org ^5^Wouter W. de Herder, MD, PhD; Erasmus MC & Erasmus MC Cancer Institute, Rotterdam, The Netherlands; w.w.deherder@erasmusmc.nl ^6^Daniel Halperin, MD; The University of Texas MD Anderson Cancer Center, Houston, TX, United States; DMHalperin@mdanderson.org ^7^Josh Mailman, MBA; Northern California CarciNET Community, Oakland, CA, United States; josh@norcalcarcinet.org ^8^Lisa Hellström, MSc; Camurus AB, Lund, Sweden; Lisa.Hellstrom@caurus.com ^8^Hanna Liedman, PhD; Camurus AB, Lund, Sweden; Hanna.Liedman@camurus.com ^8^Agneta Svedberg, MSc, MBA; Camurus AB, Lund, Sweden; Agneta.Svedberg@camurus.com ^8*^Fredrik Tiberg, PhD; Camurus AB, Lund, Sweden; Fredrik.Tiberg@camurus.com
Name and contact information for the trial sponsor	Camurus AB, Ideon Science Park, 223 70 Lund, Sweden; info@camurus.com
	This trial is funded by Camurus AB, who are responsible for the trial design as well as management, analysis and interpretation of data. Support for third-party writing assistance for this article, provided by Costello Medical, UK, was funded by Camurus AB in accordance with Good Publication Practice (GPP3) guidelines (http://www.ismpp.org/gpp3 ).
	*Correspondence to Fredrik Tiberg, PhD; Camurus AB, Lund, Sweden; Fredrik.Tiberg@camurus.com

## Introduction

### Background and rationale

Neuroendocrine tumours (NET) are heterogeneous neoplasms that account for approximately 0.5% of all newly diagnosed malignancies, with ~20% of patients with NET presenting with metastases, and a further 38% developing them after initial diagnosis [1–3].

Approximately 55–70% of NET arise from the gastrointestinal tract, as well as the pancreas, and are termed gastroenteropancreatic (GEP)-NET [3]. About 10% of GEP-NET may be functional due to hormonal/peptide hypersecretion, which is associated with debilitating symptoms, including flushing and severe secretory diarrhoea (carcinoid syndrome), bronchospasm and fibrotic heart valve disease (carcinoid heart disease) [4]. The survival of patients with GEP-NET depends on the primary tumour site; the median overall survival (OS) is 3.6 years for pancreatic NET and 8.6 years for metastatic small bowel NET [5, 6]. Aligned with this, surgical intervention is often not curative for GEP-NET, as metastases are commonly observed before or shortly after diagnosis [1].

According to international guidelines, the current standard of care (SoC) for the initial treatment of unresectable or metastatic well-differentiated GEP-NET requires initiation of first-generation somatostatin receptor ligand (SRL) therapy, octreotide and lanreotide [7, 8]. Octreotide and lanreotide are synthetic long-acting analogues of the natural inhibitory hormone somatostatin that suppress pituitary, pancreatic, biliary, gastric and intestinal secretions [2, 9]. These treatments were established as first-line agents for low-grade NET following the PROMID (octreotide) and CLARINET (lanreotide) clinical trials, having demonstrated significantly prolonged progression-free survival (PFS) compared to placebo [10–13]. Octreotide is available in immediate release (IR) and long-acting release (LAR) formulations and lanreotide is available in sustained release and saturated autogel (ATG) formulations [9, 14].

Disease progression can occur despite SoC SRL treatment, and the optimal approach to managing progression when taking first-line SRL remains undefined [2, 15, 16]. To investigate the effects of increasing SRL dosing frequency, the prospective, single-arm CLARINET FORTE trial evaluated lanreotide ATG administration at a more frequent dosing interval of every 2 weeks (Q2W) in patients whose disease had progressed at the standard dosing interval of every 4 weeks (Q4W) [17]. In patients with Ki-67 ≤10%, median PFS reported was 8.6 months (95% confidence interval [CI]: 5.6–13.8 months) for patients with mid-gut NET, and 8.0 months (95% CI 5.6–8.3 months) for patients with pancreatic NET [17]. Moreover, the control arm of the NETTER-1 trial provided further information on high-dose octreotide LAR treatment (60 mg Q4W) in patients with mid-gut NET whose disease had progressed on SoC octreotide LAR, reporting a median PFS of 8.4 months [12]. However, an analysis of 18 studies evaluating high-dose octreotide or lanreotide in GEP-NET reported variable response rates (0–14%), and considerable heterogeneity in rates of biochemical control (27–100%) or symptom control (23–100%) [15]. Prospective, randomised clinical trial data are, therefore, required to clarify the efficacy of increased SRL exposure.

The novel octreotide subcutaneous (SC) depot, CAM2029, is a high-exposure, long-acting, slow-release formulation, developed to address the limitations of current long-acting SRL [14]. CAM2029 contains the same active ingredient as octreotide LAR and octreotide IR, which have well-characterised efficacy and safety profiles [10, 11]. Previous clinical trials have shown that octreotide has greater bioavailability when formulated as CAM2029 than as octreotide LAR, without added toxicity [14, 18]. Additionally, CAM2029 administration can be undertaken by the patient or carer themselves using a pre-filled pen, whereas administration of octreotide LAR requires medical assistance; administration of lanreotide ATG also requires medical assistance in the USA, while in some regions such as the EU, a healthcare professional can decide that patients on a stable dose may self-administer or have lanreotide ATG administered by a trained person after appropriate training – the relevant references are:

Lanreotide ATG:

US FDA label – https://www.accessdata.fda.gov/spl/data/3cbf9cb7-f01-4062-9e57-43e0f1c267a1/3cbf9cb7-cf01-4062-9e57-43e0f1c267a1.xml

SmPC – https://www.hpra.ie/img/uploaded/swedocuments/Licence_PA0869-004-002_21082023113237.pdf

Octreotide LAR:

US FDA label – https://www.accessdata.fda.gov/spl/data/09e3ff5f-554a-4922-9478-d87d52e842d0/09e3ff5f-554a-4922-9478-d87d52e842d0.xml

SmPC – https://www.hpra.ie/img/uploaded/swedocuments/Licence_PA0896-028-004_03012024152159.pdf

For patients who receive a stable dose of lanreotide ATG, the product can be administered either by the patient or by a trained person (Reference Somatuline Autogel 60 mg, solution for injection in a prefilled syringe - Summary of Product Characteristics (SmPC) - (emc) (medicines.org.uk)) [14]. As 35% of patients reported the experience of lanreotide ATG treatment being dependent on the administrator as an unfavourable attribute, it will be important to explore the patient perspective of CAM2029 self-injection treatment [19]

There is a need for prospective trial data that confirm the efficacy and tolerability of escalated doses of SRL in patients with GEP-NET. The SORENTO trial aims to determine whether CAM2029 prolongs PFS compared to Investigator’s choice of SoC comparator (octreotide LAR or lanreotide ATG) [20].

## Methods

### Objectives

The primary objective of the SORENTO trial is to demonstrate superiority of treatment with CAM2029 compared to treatment with octreotide LAR or lanreotide ATG on PFS in patients with unresectable/metastatic and well-differentiated GEP-NET.

Other objectives of the trial are to evaluate and compare the two treatment groups with respect to OS, overall response rate (ORR), disease control rate (DCR) and time to/duration of tumour response (according to RECIST 1.1). In addition, the trial aims to evaluate and compare the two treatment groups with regard to patient-reported outcomes (PROs) for health-related quality of life (QoL) and patient treatment satisfaction. Finally, the trial aims to evaluate rescue medication requirements for symptom control and to describe the effect of increased dose frequency with CAM2029 in the open-label extension (OLE) period. A full list of trial objectives is provided in Table S1 of the Supplementary information section.

### Trial design

SORENTO is a prospective, multicentre, randomised, active-controlled, open-label, parallel-group, phase 3 trial. As a multicentre trial, SORENTO is anticipated to include trial sites in 11 countries across North America, Europe and Asia [20]. A detailed list of trial sites (including current recruitment status) is provided in the Supplementary information section. This trial is designed to compare treatment with 20 mg CAM2029 Q2W to treatment with the Investigator’s choice of comparator (intramuscular [IM] octreotide LAR 30 mg or SC lanreotide ATG 120 mg Q4W) in patients with advanced (unresectable or metastatic), well-differentiated NET of GEP or presumed GEP origin, using a head-to-head, superiority trial design.

**Fig. 1:**
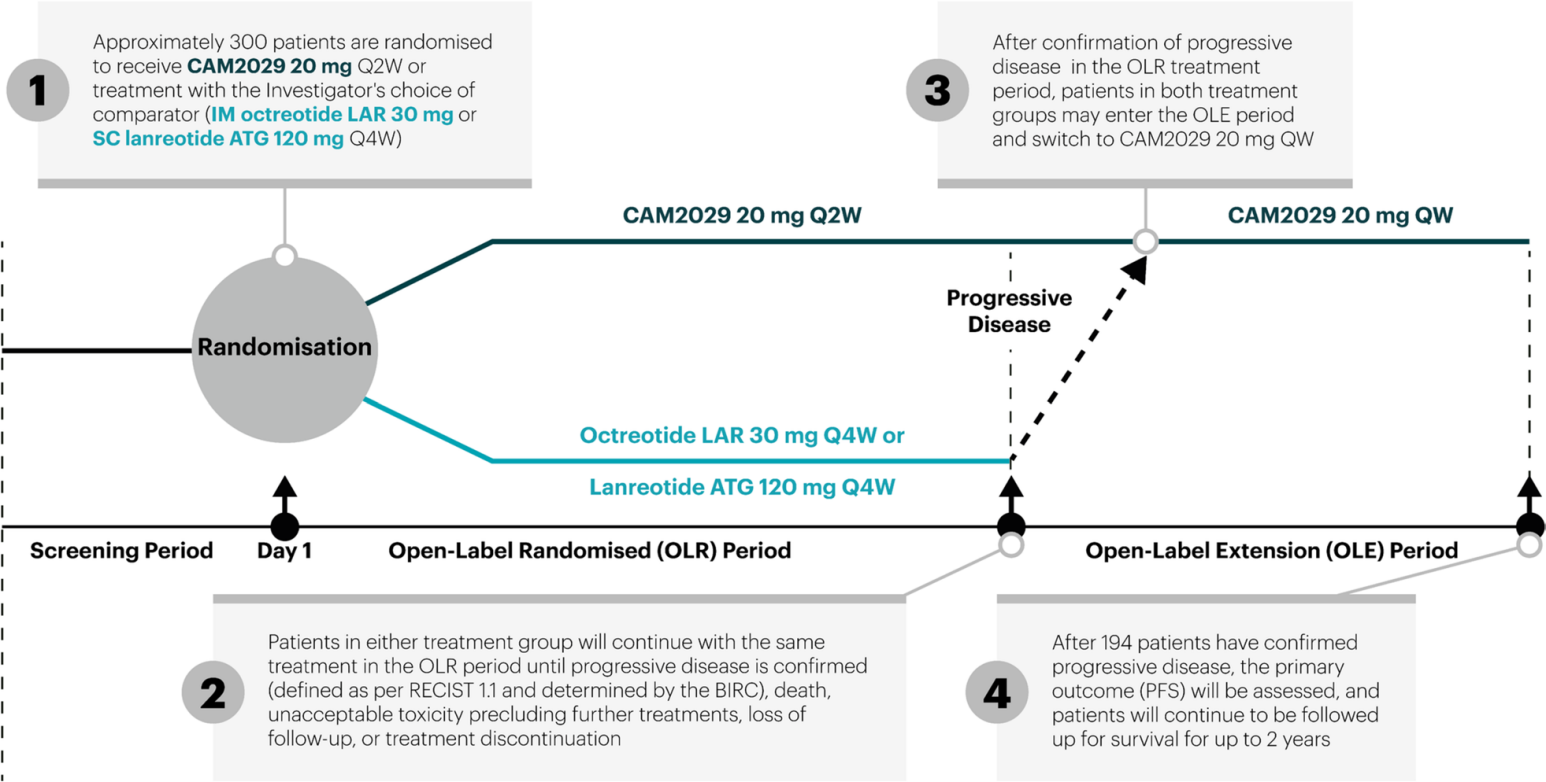
SORENTO trial design. Abbreviations: *ATG* Autogel, *BIRC* Blinded Independent Review Committee, *IM* intramuscular, *LAR* long-acting release, *mg* milligram, *OLE* open-label extension, *OLR* open-label randomised *PFS* progression-free survival *QW* once weekly *Q2W* every 2 weeks *Q4W* every 4 weeks *SC* subcutaneous

Patients will be randomised in a 1:1 ratio, stratified based on the tumour Ki-67 index (Ki-67 <10% versus Ki-67 ≥10%) [21, 22], site of tumour origin (pancreas versus other gastrointestinal origin) and choice of comparator product (octreotide LAR or lanreotide ATG). Patients in either treatment group will continue with the same treatment in the open-label randomised (OLR) treatment period until disease is confirmed as progressive (defined as per RECIST 1.1). Patients with progressive disease may then enter an optional OLE treatment period and receive 20 mg CAM2029 once weekly (QW), to investigate the effects of higher frequency dosing (Fig. 1). After the primary PFS analysis (assessed when 194 patients have confirmed progressive disease), secondary analyses will be performed according to the pre-defined test order. The trial will then continue, and the OS follow-up will be done at the latest 2 years after the primary PFS analysis. If the trial meets its primary objective, patients with ongoing comparator treatment in the OLR treatment period at time of the primary PFS analysis may switch regimen to 20 mg CAM2029 Q2W.

### Eligibility criteria

To be considered eligible to participate in the trial, patients must be ≥18 years old and have histologically confirmed, advanced (unresectable and/or metastatic), and well-differentiated NET of GEP or presumed GEP origin. They must also have ≥1 measurable, somatostatin receptor-positive (by nuclear imaging) lesion (according to RECIST 1.1) determined by multiphasic computed tomography (CT) or magnetic resonance imaging (MRI), performed within 28 days before randomisation. Patients must score between 0 and 2 on the Eastern Cooperative Oncology Group (ECOG) performance scale [23].

Patients will be excluded from the trial if they have documented evidence of disease progression whilst on treatment (including SRL) for locally advanced unresectable or metastatic disease, known central nervous system metastases or consecutive treatment with long-acting SRL for >6 months before randomisation. In addition, patients with carcinoid symptoms refractory to treatment with conventional doses of octreotide LAR or lanreotide ATG and/or to treatment with daily doses of ≤600 μg of octreotide IR will be excluded from the trial. Patients who have had prior treatment with >1 cycle of targeted therapies (e.g. mammalian target of rapamycin/vascular endothelial growth factor inhibitors); >1 cycle of chemotherapy or interferon; trans-arterial chemoembolisation or trans-arterial embolisation within 12 months before screening; or peptide receptor radionucleotide therapy (PRRT) at any time, will also be excluded. Full details of patient eligibility criteria are provided in the Supplementary information section.

### Interventions

CAM2029 will be administered as an SC injection via a ready-to-use pre-filled pen in the abdomen, thigh or buttock, as per the patient/physician choice. Patients may self-administer or have CAM2029 administered by a caregiver after appropriate training under the supervision of adequately trained personnel. Additionally, patients may administer CAM2029 at home after three successful self-administered doses. Patients administering CAM2029 at home will be expected to record doses and dates of administration in a provided diary.

Lanreotide ATG 120 mg, formulated as a pre-filled syringe, will be administered by a healthcare professional (HCP) via deep SC injection in the buttock or thigh as per local practice and regulation. Octreotide LAR 30 mg (in powder and solvent for suspension for injection) will be administered by an HCP via an IM injection in the buttock as per local practice and regulation.

All patients may receive octreotide IR (up to 600 μg per day) as a rescue medication, according to local practice. Rescue medication should be avoided within 24 h before a clinic visit.

After confirmation of progressive disease by the Blinded Independent Review Committee (BIRC) in the OLR treatment period, patients in both treatment groups may enter the OLE treatment period and switch to 20 mg CAM2029 QW. Rescue medication may continue to be used during the OLE treatment period.

Treatment will be discontinued in events such as fulfilment of Common Terminology Criteria for Adverse Events (CTCAE) Grade ≥3 (severe) adverse drug reactions that are not resolved or improved within 28 days; pregnancy; and fulfilment of hepatic-, cardiac- or hyperglycaemia-related discontinuation criteria.

### Concomitant medication

Treatments for adverse events (AEs) and symptom management (for cancer or concurrent diseases) are permitted, such as anti-emetics, pancreatic enzymes, pain medications and anti-diarrhoeal medications. Dose adjustment of medicinal products such as beta-blockers and antidiabetics or agents to control electrolyte balance may be necessary during the trial. Palliative radiation to non-target bone lesions is permitted if performed solely for bone pain relief. Bisphosphonate therapy will be permitted for the treatment of osteoporosis and the prevention of skeletal-related events in cases of bone metastases. Anti-neoplastic therapy, targeted therapies, PRRT and interferon are not permitted until the end-of-treatment/end-of-extension-treatment visit. Other investigational therapies are not permitted until survival follow-up. Treatments with a known risk of Torsades de Pointes are prohibited from 7 days or 5 half-lives prior to commencing trial treatment, up until the safety follow-up visit.

### Outcomes

#### Efficacy outcomes

The primary outcome measure will be PFS (assessed by a BIRC) from date of randomisation until date of first documented disease progression (as per RECIST 1.1) or death due to any cause [24].

Other key outcome measures include OS from the date of randomisation to the date of death due to any cause (up to 2 years post-primary efficacy analysis), ORR (defined as the proportion of patients with a best overall response of complete response [CR] or partial response [PR] as per BIRC according to RECIST 1.1, assessed at the primary endpoint analysis) and DCR (defined as proportion of patients with a best overall response of CR, PR or stable disease, as per BIRC according to RECIST 1.1, assessed at the primary endpoint analysis). Other outcome measures include time to tumour response (defined as the time from date of randomisation until first documented CR or PR, as per BIRC according to RECIST 1.1.), duration of response (defined as the time from first documented CR or PR until disease progression or death due to underlying cancer, as per BIRC according to RECIST 1.1.) and the average number of injections of rescue medication per month during the trial. In addition, outcomes include PFS-ext (defined as the time from date of randomisation to the date of documented disease progression, as per BIRC according to RECIST 1.1, or death, occurring in the OLE treatment period) and PROs (including health-related QoL, patients’ satisfaction with their medication during the trial, and patients’ experience with the trial and trial treatment in a telephone exit interview). A full list of PRO instruments include: Short Form-36 Survey (SF-36) [25], global health status/QoL scale score European Organization for Research and Treatment of Cancer Core Quality of Life Questionnaire (EORTC QLQ-C30) [26], QoL Questionnaire – Neuroendocrine Carcinoid Module (QLQ-GINET21) [27], patient satisfaction as measured by the Treatment Satisfaction Questionnaire for Medication (TSQM) [28] and Patient Global Impression of Severity (PGI-S) [29].

Of note, QLQ-GINET21 has been validated in patients with gastrointestinal NET (Cronbach’s *α*: >0.7 for all parts of the QLQ-GINET21 at 6 months; intraclass correlation: >0.85) [27]. A full list of trial outcomes is provided in the Supplementary information section.

#### Safety outcomes

Safety outcomes include treatment-emergent AE (TEAE) incidence from the first dose of CAM2029/comparator to the Safety Follow-up; changes in laboratory values, vital signs and electrocardiogram (ECG) readings, and gallbladder ultrasounds conducted every 6 months, if clinically indicated.

AEs and serious adverse events (SAEs) will be assessed from the time of signing the informed consent form and followed until a final outcome or to the Safety Follow-up visit (whichever occurs first). SAEs are defined as untoward events at any dose, resulting in (risk of) death, in-patient hospitalisation or prolongation of existing hospitalisation, significant disability, congenital anomaly or birth defect, or another medically important event. SAEs will be reported within 24 h of identification.

Safety assessments will be conducted at every visit, with AEs/SAE assessed for severity (the measure of AE or SAE intensity, according to the CTCAE). SAEs and Grade 3 (or severe) and non-serious AEs that are determined as “possibly” or “probably” related to CAM2029 or comparator treatment and are ongoing at the Safety Follow-up should be followed up on a regular basis, as per the Investigator’s judgement until an outcome is established. In the event of spontaneous reporting by the patient post-Safety Follow-up (post-trial drug-related SAEs), the Investigator will report events of this description to the Sponsor.

### Participant timeline

An overview of the participant timeline is provided in Fig. 1 and the Schedule of Assessments in Table 1 (OLR treatment period) and Table 2 (OLE treatment period). Randomisation will occur on day 1 of month 1 of the OLR treatment period. Patients will continue in the OLR treatment period until disease progression (confirmed by the BIRC), death, unacceptable toxicity precluding further treatments, loss to follow-up or treatment discontinuation determined by the patient or Investigator.

**Table 1:**
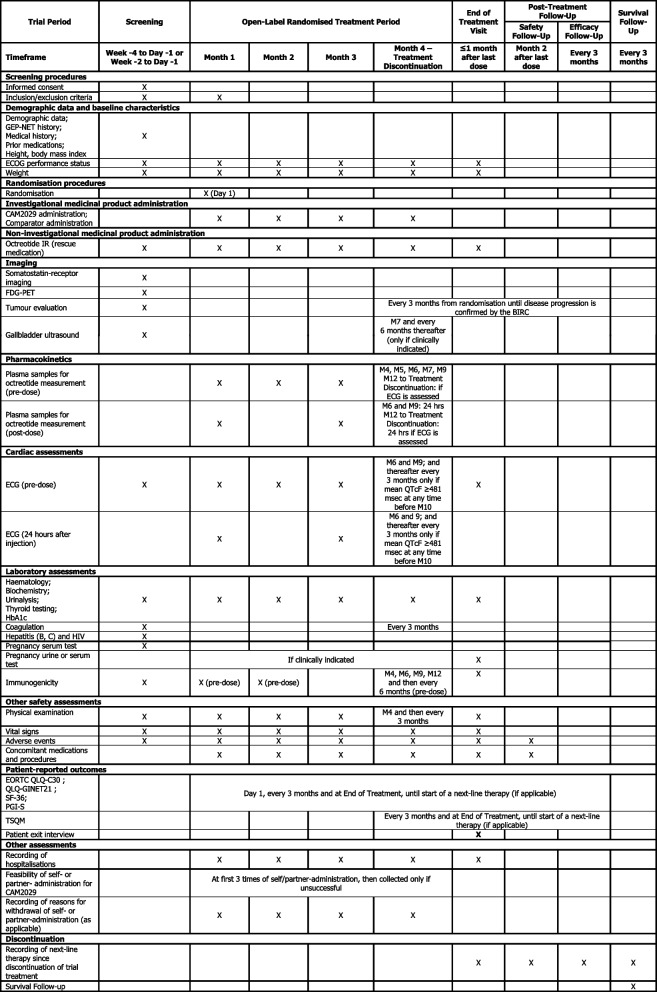
Schedule of trial procedures and assessments for the open-label randomised treatment period and the post-treatment and survival follow-up periods

*Abbreviations*: *BIRC* Blinded Independent Review Committee, *ECG* electrocardiogram, *ECOG* Eastern Cooperative Oncology Group, *EORTC QLQ-C30* European Organization for Research and Treatment of Cancer Core Quality of Life Questionnaire, *FDG* fluorodeoxyglucose, *GEP-NET* gastroenteropancreatic neuroendocrine tumours, *HbA1c* haemoglobin A1c, *HIV* human immunodeficiency virus, *hrs* hours, *IR* immediate release, *M* month, *msec* millisecond, *PET* positron emission tomography, *PGI-S* Patient Global Impression of Severity, *QLQ-GINET21* Quality of Life Questionnaire – Neuroendocrine Carcinoid Module, *QTcF* QTc interval corrected by Friderica’s formula, *SF-36* Short Form-36 Survey, *TSQM* Treatment Satisfaction Questionnaire for Medication

**Table 2:** Schedule of trial procedures and assessments for patients participating in the optional OLE treatment period

Trial period	Open-label extension treatment period		End-of-extension treatment visit	Post-treatment follow-up		Survival follow-up
				Safety follow-up	Efficacy follow-up	
Timeframe	Extension month 1	Extension month 2 – treatment discontinuation	≤1 month after last dose	Month 2 after last dose	Every 3 months	Every 3 months
**Eligibility procedures**						
Inclusion/exclusion criteria for the OLE treatment period	X					
**Demographic data and baseline characteristics**						
ECOG performance status	X	ME2, ME3 and thereafter every 3 months				
Weight	X	ME2, ME3 and thereafter every 3 months	X			
**Investigational medicinal product administration**						
CAM2029 QW	X	X				
**Non-investigational medicinal product administration**						
Octreotide IR (rescue medication)	X	X	X			
**Imaging**						
Tumour evaluation	Every 3 months from randomisation until new disease progression is confirmed by the BIRC					
Gallbladder ultrasound		Every 6 months (only if clinically indicated)				
**Pharmacokinetics**						
Plasma samples for octreotide measurement (pre-dose)	X	ME2, ME3				
Plasma samples for octreotide measurement (24 hrs after injection)		ME2, ME3 and ME6: 24 hrs ME9 to Treatment Discontinuation: 24 hrs if ECG is assessed				
**Cardiac Assessments**						
ECG (pre-dose)	X	ME2, ME3	X			
ECG (24 hrs after injection)		ME2, ME3, ME6 and thereafter every 3 months only if mean QTcF ≥481 msec at any time before ME6				
**Laboratory assessments**						
Haematology; Biochemistry; Urinalysis; Thyroid testing; HbA1c	X	ME2, ME3 and thereafter every 3 months	X			
Coagulation	X	ME3 and thereafter every 3 months				
Pregnancy urine or serum test	X	If clinically indicated	X			
Immunogenicity	X (pre-dose)	Every 6 months (pre-dose)	X			
**Other safety assessments**						
Physical examination; Vital signs	X	ME2, ME3 and thereafter every 3 months	X			
Adverse events	X	X	X	X		
Concomitant medications and procedures	X	X	X	X		
**Patient-reported outcomes**						
EORTC QLQ-C30; QLQ-GINET21; SF-36; PGI-S; TSQM	ME1D1 ME3D1, every 3 months from ME3D1 and at End of Extension Treatment, until start of a next-line therapy (if applicable)					
**Other assessments**						
Recording of hospitalisations	X	X	X			
Feasibility of self or partner-administration	At first 3 times of self/partner-administration, then collected only if unsuccessful					
Recording of reasons for withdrawal of self- or partner-administration (as applicable)	X	X				
**Discontinuation**						
Recording of next-line therapy since discontinuation of IMP			X	X	X	X
Survival Follow-up						

*Abbreviations*: *BIRC* Blinded Independent Review Committee, *ECG* electrocardiogram, *ECOG* Eastern Cooperative Oncology Group, *EORTC QLQ-C30* European Organization for Research and Treatment of Cancer Core Quality of Life Questionnaire, *HbA1c* haemoglobin A1c, *hrs* hours, *IMP* investigational medicinal product, *IR* immediate release, *ME* Month in Extension Period, *MED* Month in Extension Period Day, *msec* millisecond, *OLE* open-label extension, *PGI-S* Patient Global Impression of Severity, *QLQ-GINET21* Quality of Life Questionnaire – Neuroendocrine Carcinoid Module, *QTcF* QTc interval corrected by Friderica’s formula, *QW* once weekly, *SF-36* Short Form-36 Survey, *TSQM* Treatment Satisfaction Questionnaire for Medication

Patients who are radiologically determined to have progressive disease according to RECIST 1.1 will undergo expedited tumour response review by the BIRC. If progressive disease is unconfirmed by the BIRC, patients should continue treatment (if clinically acceptable) until disease progression is confirmed. If patients from either treatment group discontinue the OLR treatment period due to progressive disease, they may begin treatment with 20 mg CAM2029 QW in the OLE treatment period. Patients who discontinue CAM2029/comparator treatment for reasons other than progressive disease will attend efficacy follow-up visits every 12 weeks until the BIRC confirms disease progression, or the patient withdraws consent, dies or is lost to follow-up. Patients discontinuing treatment or exiting the efficacy follow-up period will enter the survival follow-up (assessed every 12 weeks for survival status and disease progression on subsequent anti-neoplastic therapies [progression on next-line therapy]). This will continue until consent withdrawal, death, or the patient is lost to follow-up.

### Recruitment and assignment of interventions

Patient recruitment methods are at the discretion of the trial sites; all sites will maintain a screening log of all screened patients. Potential eligible patients will be selected as outlined in the Eligibility Criteria section, and selected patients will be informed in detail about the trial purpose and benefits, procedures/interventions and any possible side effects.

Eligible patients will be randomised in a 1:1 ratio to CAM2029 or active comparator using an interactive randomisation system. The Investigator will prospectively choose either octreotide LAR or lanreotide ATG as the potential comparator drug before the patient is randomised into the trial.

To ensure balance between treatment groups in key factors that could potentially impact PFS, and to account for a potential difference in comparator efficacy, randomisation will be stratified, using permuted blocks, by the following:

- Histological grade of nuclear antigen Ki-67 <10% versus Ki-67 ≥10%
- Tumour origin (pancreas versus any other gastrointestinal origin)
- Intended choice of comparator (octreotide LAR or lanreotide ATG)

In this open-label, randomised, controlled trial, a BIRC will determine the presence of progressive disease and address any potential sources of bias from the inherent subjective component of PFS assessment, as per RECIST 1.1.

#### Sample size

Sample size calculations were performed in PASS 16® (NCSS Statistical Software), assuming uniform patient accrual, and without adjustment for potential loss to follow-up or for treatment crossover. Sample size assumptions are based on 18 months for recruitment and 30 months of follow-up, i.e. a total of 48 months. At least 194 events are needed to achieve the desired statistical power for the primary outcome, corresponding to 280 patients. In order to account for dropouts, approximately 300 patients are planned to be enrolled in the trial. A one-sided log rank test with an overall sample size of 280 patients (140 in the CAM2029 treatment group and 140 in the comparator treatment group) will achieve at least 85% power at a 0.025 (one-sided) significance level to detect a potential hazard ratio of 0.65 when the comparator group median time to progression or death is 18 months.

### Statistical methods

All efficacy analyses will be conducted based on the intention-to-treat analysis (ITT) set, which comprises all patients randomised to a treatment group.

#### Primary endpoint

The primary efficacy endpoint (PFS in the OLR treatment period, determined by the BIRC, as per RECIST 1.1) will be analysed according to treatment group. The hazard ratio and associated 95% CI will be estimated using a Cox regression model, with stratification factors the same as those used in randomisation, and censoring will occur at the date of final tumour assessment if an event does not occur prior to the date of analysis cut-off. Sensitivity analyses and informative censoring will be used to interrogate the determination of this primary efficacy endpoint, in which different methods for handling intercurrent events and different assumptions for missing data will be explored. A per-protocol analysis will also be conducted on the per-protocol analysis set (all patients in the ITT analysis set with no major protocol deviations that would impact the efficacy assessment).

An interim analysis for OS will be performed in connection to the primary PFS analysis.

#### Secondary endpoints

OS and time to tumour response will be analysed as secondary endpoints, alongside PFS-ext as an exploratory endpoint, using a similar model as for the primary efficacy analysis. Superior CAM2029 efficacy, based on improvements in OS, will be declared at the end of the OLR treatment period only if robust efficacy is demonstrated. Assuming that OS will be long-lasting in the trial population, the final analysis of OS will be time-driven and not event-driven. If the trial continues to the final analysis (approximately 2 years after primary analysis), the alpha level that will be used to declare statistical significance at this final analysis will be 0.0226 (one-sided) based on an O’Brien-Fleming test, where 0.0072 is first allocated to the interim analysis for OS. The ORR and DCR will be analysed separately using a Cochran-Mantel-Haenszel test stratified by the randomisation stratification factors. Duration of response will be summarised by descriptive statistics for the best response of PR and CR. Patients without disease progression/death due to any cause will be censored at the date of the last tumour assessment.

Standard scoring algorithms will be applied for derivation of dimensions/domains for each PRO, as described by the developer. Mixed models for repeated measures will be used to assess change from baseline in the PRO endpoints, which accounts for missing data under missing at random (MAR). Rescue medication use (total dosage, dose intensity) will be described by summary statistics.

#### Safety endpoint analyses

Based on the safety analysis set (all patients administered at least 1 dose of CAM2029 or comparator treatment), TEAEs will be evaluated according to severity, relationship, outcome and seriousness by Medical Dictionary for Regulatory Activities system organ class and preferred term. TEAEs with an incidence of ≥5% (or lower threshold applicable for reporting) will also be presented. Changes in laboratory values, vital signs and ECG readings will be summarised using standard statistical analyses for continuous data, and categorical data will be presented in shift tables. Triplicate 12-lead ECGs, including ECG intervals, will be evaluated descriptively.

### Data collection and management

The imaging assessment plan for efficacy analysis includes somatostatin-receptor imaging (during the screening period), multiphasic CT or MRI and fluorodeoxyglucose positron emission tomography (FDG-PET; encouraged for patients with Grade 3 NET during the screening period only). In addition, whole-body bone scan, brain CT or MRI and additional imaging may be performed as clinically indicated. Radiologic evidence of progressive disease will be verified by the BIRC at the central imaging core laboratory following determination by the local Investigator.

Investigator personnel will be trained in patient retention and preventing data loss, and the Investigator will document all cases of discontinuation, including justifications for discontinuation. Patients who discontinue treatment with CAM2029 or comparator should not be considered withdrawn from the trial and should instead return for the assessments indicated in Tables 1 and 2; if they fail to return for these assessments for unknown reasons, every effort (e.g. telephone, email, letter) should be made to contact them. Whilst all possible measures should be made to report the reasons for patient-led withdrawal, this will not be an obligation for patients.

An electronic case report form system is used for data capture, except for external data (e.g. ECG and clinical laboratory results, and tumour response data from the BIRC), which may be transferred electronically. Patients will not be identified by their names, but by their screening or randomisation number. Functions, processes and specifications for data collection cleaning and validation will be documented in a data management plan.

Following trial withdrawal, all previously collected results for that patient may be retained and used for trial evaluation. This includes retention of biological samples until trial completion and reporting (or as by local standards). Additional collection of biological specimens is not planned for use in future ancillary studies.

### Oversight and monitoring

The steering committee includes authors SS, DF, JC, JAC, WWdH, DH and JM and is an advisory committee participating in the planning and oversight of the trial to provide support and expert advice and recommendations during the design, conduct and reporting of the clinical trial. JM is a patient representative providing insights and experience on all aspects of the trial. Additional members of the steering committee include Simona Grozinsky-Glasberg (Neuroendocrine Tumor Unit, ENETS Center of Excellence, Hadassah Medical Organization and Faculty of Medicine, Hebrew University, Jerusalem, Israel) and Thorvardur Halfdanarson, MD (Mayo Clinic Comprehensive Cancer Center, Rochester, MN, United States). SS serves as the international coordinating investigator for the trial and the chair of the steering committee. The data monitoring committee (DMC) includes a minimum of one statistician and two physicians and is responsible for periodic assessments of trial safety data, continuous review of Grade 3/serious adverse drug reactions and making recommendations to the Sponsor where applicable. Members of the DMC will not be involved in other trial-related tasks and have no competing interests.

## Discussion

SORENTO is expected to be the first trial to investigate the efficacy of a high-exposure SRL versus the SoC with first-generation long-acting treatments (octreotide LAR and lanreotide ATG) in patients with GEP-NET using a head-to-head, superiority trial design. This will allow high-quality and robust collection of data to provide meaningful treatment comparisons. To date, studies investigating SRL dose escalation in this patient population have largely been retrospective, whilst those that were prospective have lacked a comparator group or included much smaller patient cohorts [10, 13, 17, 30]. The SORENTO trial will, therefore, provide key insights into the feasibility of increased SRL dosing treatment strategies.

Furthermore, the primary endpoint (PFS) will be determined by a BIRC. The BIRC will allow for assessments to be made without knowledge of treatment assignment and can reduce potential systematic imaging reader bias, as well as measurement variability [31]. The BIRC will also assess endpoints for intensified treatment with CAM2029 in patients who progressed to the OLE treatment period. This will ensure high-quality efficacy data are obtained for high-exposure CAM2029 in patients who experience disease progression during SoC SRL treatment.

Another key strength of this trial design is its inclusion of patients with well-differentiated Grade 3 NET. Patients with well-differentiated Grade 3 NET should have a FDG positron emission tomography (PET), with all FDG positive lesions being somatostatin receptor (SSTR) positive. The definition of this subpopulation was introduced in the 2017 World Health Organization (WHO) grading system classification, following observations that this subgroup typically had better prognosis than patients with poorly differentiated neuroendocrine carcinomas [32]. Subsequently, therapeutic data for this specific subpopulation remains scarce and there is a clear unmet need for treatment alternatives for these patients [33]. Where many studies in this field lack reliable differentiation of Grade 3 GEP-NET, this patient group will be included in the SORENTO trial, which may provide important information for the treatment strategy of this population [33].

Additionally, for patients receiving CAM2029, SORENTO will allow for successfully trained trial participants to self-administer the treatment, providing potential practical and financial benefits to patients, carers and HCPs. For patients, this would increase treatment autonomy and may improve QoL. During the COVID-19 pandemic, there has been a significant shift in clinical trial management to allow for self-isolation, with remote trials becoming more commonplace [34]. Decentralised trials have been associated with increased patient empowerment and patient-centred care [35], and it has been suggested that the convenience of home administration may improve patient retention and compliance [36, 37].

Given the range of administration options of available treatments in GEP-NET, it will be important to capture the patient perspective around treatment administration preferences, as well as information on the impact of the intervention more generally. The trial will use a variety of PROs, including both generic and disease-specific questionnaires to establish the patient perspective, e.g. the TSQM questionnaire to measure patient satisfaction with the medicine, QoL questionnaires and patient exit interviews to provide key insights into benefits and challenges associated with treatment options [26–28].

The open-label design of this trial introduces a potential source of bias. This bias is limited by using a BIRC for the objective tumour response evaluation, including confirmation of disease progression for the primary endpoint. In addition, both treatment groups will be treated and assessed in a similar way, irrespective of their assigned treatment, except for patients receiving CAM2029 who will have the option to self-administer from home. Furthermore, the design of the trial is warranted as the frequency and modality of the two treatment groups are different. Patients self-administering CAM2029 will still attend monthly clinic visits (i.e. the same frequency as patients receiving comparator treatment), to ensure the nature and timing of assessments are aligned between the trial treatment groups.

In addition, due to OS not being a feasible endpoint in NET, PFS is the generally accepted primary treatment target in NET and, therefore, is used as the primary outcome of this trial [38]. It should also be noted that the use of PFS as the primary endpoint may lead to the potential for informative censoring, for example if patients experience clinical benefit and discontinue from the trial treatment (and are censored as a result), they may be less likely to experience disease progression as a sub-population than non-censored patients. However, patients will be followed-up regardless of treatment discontinuation, until the clinical event of interest is observed, the trial ends, or the patient is lost to follow-up, in order to mitigate against non-informative censoring [39].

## Trial status

The trial protocol was approved by the independent ethics committee (IEC)/institutional review board (IRB) prior to the trial start date. The trial began in October 2021, and the first patient was randomised in November 2021. The trial included 89 study locations in 11 countries, as of April 2023. Recruitment will be completed during 2023.

### Supplementary Information

**Additional file 1: Table S1.** Supplementary Information.

**Additional file 2. **SPIRIT Checklist.

## Data Availability

The results will be published in a peer-reviewed journal after the trial ends. No personal information about any of the participants will be disclosed. All available trial-related source data and records will be made accessible to the mandated auditor(s), or to domestic/foreign regulatory inspectors or representatives from IECs/IRBs who may audit/inspect the trial.

## Abbreviations

AE: Adverse event
ATG: Autogel
BIRC: Blinded Independent Review Committee
CI: Confidence interval
CR: Complete response
CT: Computed tomography
CTCAE: Common Terminology Criteria for Adverse Events
DCR: Disease control rate
DMC: Data monitoring committee
ECG: Electrocardiogram
ECOG: Eastern Cooperative Oncology Group
EORTC QLQ-C30: European Organisation for Research and Treatment of Cancer Core Quality of Life Questionnaire
FDG: Fluorodeoxyglucose
GEP-NET: Gastroenteropancreatic neuroendocrine tumours
GPP3: Good Publication Practice 3
HbA1c: Haemoglobin A1c
HCP: Healthcare professional
HIV: Human immunodeficiency virus
IEC: Independent ethics committee
IM: Intramuscular
IMP: Investigational medicinal product
IR: Immediate release
IRB: Institutional review board
LAR: Long-acting release
M: Month
ME: Month in Extension Period
MED: Month in Extension Period Day
MRI: Magnetic resonance imaging
NET: Neuroendocrine tumour
OLE: Open-label extension
OLR: Open-label randomised
ORR: Overall response rate
OS: Overall survival
PET: Positron emission tomography
PFS: Progression-free survival
PGI-S: Patient Global Impression of Severity
PR: Partial response
PRO: Patient-reported outcome
PRRT: Peptide receptor radionucleotide therapy
Q2W: Every 2 weeks
Q4W: Every 4 weeks
QLQ-GINET: Quality of Life Questionnaire – Neuroendocrine Carcinoid Module
QoL: Quality of life
QW: Once weekly
SAE: Serious adverse event
SC: Subcutaneous
SF-36: Short Form-36 Survey
SoC: Standard of care
SRL: Somatostatin receptor ligand
TEAE: Treatment-emergent adverse event
TSQM: Treatment Satisfaction Questionnaire for Medication
WHO: World Health Organization
