# Correction: Methodology of the SORENTO clinical trial: a prospective, randomised, active-controlled phase 3 trial assessing the efficacy and safety of high exposure octreotide subcutaneous depot (CAM2029) in patients with GEP-NET

**DOI:** 10.1186/s13063-024-08146-1

**Published:** 2024-05-02

**Authors:** Simron Singh, Diego Ferone, Jaume Capdevila, Jennifer Ang Chan, Wouter W. de Herder, Daniel Halperin, Josh Mailman, Lisa Hellström, Hanna Liedman, Agneta Svedberg, Fredrik Tiberg

**Affiliations:** 1 Sunnybrook Health Sciences Center, Toronto, Canada; 2 Endocrinology, Department of Internal Medicine & Medical Specialties, University of Genova, Endocrinology Clinic IRCCS Ospedale Policlinico San Martino, Genoa, Italy; 3 Vall d’Hebron University Hospital, Barcelona, Spain; 4 Dana-Farber Cancer Institute, Boston, MA USA; 5 Erasmus MC & Erasmus MC Cancer Institute, Rotterdam, The Netherlands; 6 The University of Texas MD Anderson Cancer Center, Houston, TX USA; 7 Northern California CarciNET Community, Oakland, CA USA; 8 Camurus AB, Lund, Sweden

**Correction: Trials 25, 58 (2024)**

**https://doi.org/10.1186/s13063-023-07834-8**

Following publication of the original article [1], we have been notified that a correction is needed on page 3, paragraph 2 as shown below:

Original statement: “Additionally, CAM2029 administration can be undertaken by the patient or carer themselves using a pre-filled pen, whereas administration of both octreotide LAR and lanreotide ATG requires medical assistance”.

Corrected statement: • Corrected statement: “Additionally, CAM2029 administration can be undertaken by the patient or carer themselves using a pre-filled pen, whereas administration of both octreotide LAR and lanreotide ATG requires medical assistance; administration of lanreotide ATG also requires medical assistance in the USA, while in some regions such as the EU, a healthcare professional can decide that patients on a stable dose may self-administer or have lanreotide ATG administered by a trained person after appropriate training” – the relevant references are:

Lanreotide ATG:

US FDA label – https://www.accessdata.fda.gov/spl/data/3cbf9cb7-cf01-4062-9e57-43e0f1c267a1/3cbf9cb7-cf01-4062-9e57-43e0f1c267a1.xml

SmPC – https://www.hpra.ie/img/uploaded/swedocuments/Licence_PA0869-004-002_21082023113237.pdf

Octreotide LAR:

US FDA label – https://www.accessdata.fda.gov/spl/data/09e3ff5f-554a-4922-9478-d87d52e842d0/09e3ff5f-554a-4922-9478-d87d52e842d0.xml

SmPC – https://www.hpra.ie/img/uploaded/swedocuments/Licence_PA0896-028-004_03012024152159.pdf

The original article has been corrected.
