# Supplementary material for: Methodology of the SORENTO clinical trial: a prospective, randomised, active-controlled phase 3 trial assessing the efficacy and safety of high exposure octreotide subcutaneous depot (CAM2029) in patients with GEP-NET
Source: Trials. 2024 Jan 16;25:58. doi: 10.1186/s13063-023-07834-8 (PMC10790497; doi:10.1186/s13063-023-07834-8)
Supplement: Supplementary file 1 — Additional file 1: Table S1. Supplementary Information. [file 13063_2023_7834_MOESM1_ESM.docx]

Supplementary Information

Full list of trial objectives and endpoints

Table 1. Trial objectives and endpoints

| **Primary Objective** | **Primary Endpoint** |
| --- | --- |
| - To assess superiority of treatment with CAM2029 compared to treatment with octreotide LAR or lanreotide ATG on PFS in patients with unresectable/metastatic and well-differentiated GEP‑NET | - PFS, defined as the time from the date of randomisation to the date of the first documented disease progression as per RECIST 1.1 or death due to any cause, whichever occurs first, as assessed by a BIRC |
| **Secondary Objectives** | **Secondary Endpoints** |
| - To assess superiority of treatment with CAM2029 compared to treatment with octreotide LAR or lanreotide ATG with respect to PFS based on local Investigator assessment | - PFS using RECIST 1.1 as assessed by local Investigators |
| - To compare the two treatment groups with respect to OS | - OS |
| - To evaluate the two treatment groups with respect to ORR and DCR | - ORR, defined as the proportion of patients with best overall response of CR or PR as per RECIST 1.1. - DCR, defined as the proportion of patients with best overall response of CR, PR or SD as per RECIST 1.1 |
| - To describe time to tumour response and duration of response in the two treatment groups | - Time to response and duration of response as per RECIST 1.1 |
| - To evaluate the need for rescue medication for symptom control in the two treatment groups | - Average number of injections of octreotide rescue medication per month for each patient during the trial - Total dosage and dose intensity of rescue medication |
| - To assess the PK of octreotide after CAM2029 administration | - Octreotide plasma concentrations over time |
| - To assess octreotide exposure–response relationship for CAM2029 | - Correlation between octreotide concentration and other endpoints or measures as appropriate |
| - To assess supervised self- or partner-administration of CAM2029 | - Proportion of patients/partners declared competent by trial personnel to administer CAM2029 out of those trying |
| - To evaluate PROs for health-related QoL in the two treatment groups | - Change from baseline in QLQ‑GINET21, SF-36 and the global health status/QoL scale score of the EORTC QLQ-C30 |
| - To evaluate the two treatment groups with respect to patient satisfaction with CAM2029 or the comparator treatment during the trial | - TSQM scores over time using all four domains of TSQM (effectiveness, side effects, convenience, and global satisfaction) |
| - To confirm the safety and tolerability of CAM2029 in patients with unresectable/metastatic and well-differentiated GEP-NET | - AEs (including local tolerability) - Changes in laboratory values, vital signs and ECG readings |
| **Exploratory Objectives** | **Exploratory Endpoints** |
| - To describe the effect of intensified treatment with CAM2029 (20 mg QW) in the OLE treatment period | - PFS-ext, defined as time from date of randomisation to the date of documented disease progression as per RECIST 1.1 or death from any cause, whichever occurs first, in the OLE treatment period, as assessed by a BIRC |
| - To compare the two treatment groups with respect to PFS2 based on local Investigator assessment | - PFS2, defined as time from date of randomisation to the date of documented progression as per RECIST 1.1 on next-line therapy (not including CAM2029 in the OLE treatment period) or death from any cause, whichever occurs first |
| - To assess hospital resource utilisation | - Number of patients hospitalised - Total number and length of hospitalisations |
| - To evaluate the two treatment groups with respect to treatment experience | - Describe patients’ experiences with the trial regimen and trial activities using data from patient exit interviews - Explore the minimal important difference for EORTC QLQ-C30 using patient exit interviews |
| - To evaluate the two treatment groups with respect to patients’ impression of disease severity | - To explore the minimal important difference for EORTC QLQ-C30 using the PGI-S and patient exit interviews |
| - To evaluate the two treatment groups with respect to ECOG performance status | - Time to deterioration of ECOG performance status |
| - To assess the immunogenicity of octreotide in patients with metastatic/inoperable and well-differentiated GEP-NET | - Qualification and quantification of anti-octreotide antibodies |

**Abbreviations:** AE: adverse event; ATG: autogel; BIRC: Blinded Independent Review Committee; CR: complete response; DCR: disease control rate; ECG: electrocardiogram; ECOG: Eastern Cooperative Oncology Group; EORTC QLQ-C30: European Organisation for Research and Treatment of Cancer’s Core Quality of Life Questionnaire; GEP-NET: gastroenteropancreatic neuroendocrine tumours; LAR: long-acting release; OLE: open-label extension; ORR: overall response rate; OS: overall survival; PFS: progression-free survival; PFS2: progression-free survival 2; PFS‑ext: progression-free survival in the Extension Treatment Period; PGI-S: Patient Global Impression of Severity; PK: pharmacokinetics; PR: partial response; PROs: patient-reported outcomes; QLQ-GINET21: Quality of Life Questionnaire – Neuroendocrine Carcinoid Module; QoL: quality of life; QW: every week; RECIST 1.1: Response Evaluation Criteria in Solid Tumours version 1.1; SD: stable disease; SF-36: Short Form-36 Survey; TSQM: Treatment Satisfaction Questionnaire for Medication.

List of trial sites

Correct as of 26^th^ April 2023. Up to date information on recruitment status can be obtained from the ClinicalTrials.gov register (NCT05050942).

United States

- Mayo Clinic Cancer Center (MCCC) – Phoenix, Arizona, United States
- Rocky Mountain Cancer Centers – Denver – Midtown, Denver, Colorado, United States
- Mayo Clinic Hospital –Jacksonville, Florida, United States
- University of Kentucky (UK) - Markey Cancer Center, Lexington, Kentucky, United States
- East Jefferson General Hospital, Metairie, Louisiana, United States
- Dana-Farber Cancer Institute, Boston, Massachusetts, United States
- Mayo Clinic Rochester, Rochester, Minnesota, United States
- Memorial Sloan-Kettering Cancer Center, New York, New York, United States
- White Plains Hospital – Center for Cancer Care, White Plains, New York, United States
- Texas Oncology –Austin, Texas, United States
- Texas Oncology –Dallas, Texas, United States
- Texas Oncology – Denton North, Denton, Texas, United States
- The University of Texas – MD Anderson Cancer Center, Houston, Texas, United States
- Texas Oncology –McAllen, Texas, United States
- Texas Oncology – San Antonio Northeast, San Antonio, Texas, United States
- Huntsman Cancer Institute, Salt Lake City, Utah, United States

Belgium

- Cliniques Universitaires Saint-Luc, Brussels, Belgium
- Hôpital Erasme, Brussels, Belgium
- Antwerp University Hospital, Edegem, Belgium
- Algemeen Ziekenhuis Maria Middelares, Gent, Belgium
- Universitair Ziekenhuis Leuven, Leuven, Belgium
- Centre Hospitalier Chretien (CHC) – Clinique Notre-Dame – Hermalle, Liège, Belgium
- AZ Nikolaas, Sint-Niklaas, Belgium

Canada

- London Health Sciences Centre, London, Canada
- The Ottawa Hospital – General Campus, Ottawa, Canada
- Princess Margaret Cancer Centre, Toronto, Canada
- Sunnybrook Health Sciences Centre, Toronto, Canada
- BC Cancer Agency Vancouver Centre, Vancouver, Canada

France

- CHU Hopitaux de Bordeaux – Hôpital Haut-Lévêque, Bordeaux, France
- CHRU Brest – Hopital Morvan – Institut de Cancerologie et d'Hematologie, Brest, France
- CHRU de Tours - Hopital Trousseau, Chambray-lès-Tours, France
- Centre Hospitalier Universitaire Dijon Bourgogne – L'Hopital General, Dijon, France
- Groupe Hospitalier de l'Institut Catholique de Lille – Hopital Saint Vincent de Paul, Lille, France
- CHU de Lyon – Groupement Hospitalier Edouard Herriot, Lyon, France
- CHU de Nantes – Hôtel-Dieu, Nantes, France
- CHU de Poitiers, Poitiers, France
- Centre Eugène Marquis, Rennes, France
- Hôpitaux Universitaires de Strasbourg – Hôpital de Hautepierre, Strasbourg, France

Germany

- Charite – UB – CVK – Medizinische Klinik, Berlin, Germany
- Universitätsklinikum Essen, Essen, Germany
- Universitätsklinikum Hamburg-Eppendorf (UKE), Hamburg, Germany
- Nationales Centrum für Tumorerkrankungen (NCT) Heidelberg, Heidelberg, Germany
- Medizinischen Fakultät Mannheim der Universität Heidelberg, Mannheim, Germany
- Universitätsklinikum Ulm, Ulm, Germany

Hungary

- SE ÁOK I. sz. Belgyógyászati Klinika, Budapest, Hungary
- Petz Aladár Megyei Oktató Kórház, Győr, Hungary
- Bács-Kiskun Megyei Kórház, Kecskemét, Hungary
- Szegedi Tudományegyetem; I. Belgyógyászati Klinika, Szeged, Hungary

Israel

- Rambam Medical Center, Haifa, Israel
- Hadassah Medical Center (HMC) – Hadassah University Hospital (HUH) – Ein Kerem, Jerusalem, Israel
- The Chaim Sheba Medical Center, Ramat Gan, Israel
- Tel Aviv Sourasky Medical Center, Tel Aviv, Israel

Italy

- Centro di Referimento Oncologico (CRO), Aviano, Italy
- Universita degli Studi di Bari – Aldo Moro, Bari, Italy
- Azienda Ospedaliero – Universitaria di Bologna Policlinico S. Orsola – Malpighi, Bologna, Italy
- ASST degli Spedali Civili di Brescia, Brescia, Italy
- Universita degli Studi di Firenze – Azienda Ospedaliero – Universitaria Careggi – SC di Oncologia Medica, Firenze, Italy
- Universita Degli Studi di Genova – Center of Excellence for Biomedical Research (CEBR), Genova, Italy
- Istituto Scientifico Romagnolo per lo Studio e la Cura dei Tumori – IRST IRCCS, Meldola, Italy
- Istituto Clinico Humanitas, Milan, Italy
- Azienda Ospedaliero – Universitaria di Modena Policlinico, Modena, Italy
- IRCCS – Istituto Nazionale dei Tumori di Napoli Fondazione G. Pascale – Oncologia Addominale , Napoli, Italy
- Istituto Oncologico Veneto – IRCCS, Padova, Italy
- Azienda Ospedaliera Sant'Andrea, Roma, Italy
- Azienda Ospedaliera Universitaria Integrata Verona – Ospedale Borgo Trento, Verona, Italy

Netherlands

- Nederlands Kanker Instituut – Antoni van Leeuwenhoek Ziekenhuis, Amsterdam, Netherlands
- Rijnstate Ziekenhuis – Arnhem, Arnhem, Netherlands
- Maastricht UMC+, Maastricht, Netherlands
- Erasmus MC, Rotterdam, Netherlands

Romania

- Institutul Clinic Fundeni, Bucharest, Romania
- Institutul Oncologic "Prof. Dr. Ion Chiricuţă" Cluj Napoca, Cluj-Napoca, Romania
- Medisprof SRL, Cluj-Napoca, Romania
- Centrul de Oncologie Sfantul Nectarie, Craiova, Romania
- Onco Clinic Consult SA, Craiova, Romania
- Sigmedical Services S.R.L., Suceava, Romania

Spain

- Complexo Hospitalario Universitario A Coruña, A Coruña, Spain
- Hospital Universitari Vall d'Hebron – Vall d'Hebron Institut d'Oncologia (VHIO), Barcelona, Spain
- Institut Catala d'Oncologia Hospitalet , Barcelona, Spain
- Hospital General Universitario de Elche, Elche, Spain
- Facultad de Medicina - Hospital Universitario Fundacion Jimenez Diaz (UAM‑FJD) (Clinica de la Concepcion), Madrid, Spain
- Hospital Universitario Fundación Alcorcón, Madrid, Spain
- Hospital Universitario La Paz (HULP), Madrid, Spain
- Hospital Universitario Ramón y Cajal, Madrid, Spain
- MD Anderson Cancer Center – Madrid, Madrid, Spain
- Hospital General Universitario Morales Meseguer, Murcia, Spain
- Hospital Universitario Virgen de la Victoria, Málaga, Spain
- Hospital Universitario Central de Asturias, Oviedo, Spain
- Parc Taulí Sabadell Hospital Universitari, Sabadell, Spain
- Hospital Universitario Marques de Valdecilla (HUMV), Santander, Spain

Full list of trial eligibility criteria

**Inclusion criteria** – Patients meeting each of the following criteria at screening (unless otherwise specified) will be eligible to participate in the clinical trial:

- - Voluntary and valid written informed consent to participate in the trial obtained (before trial-related procedures are performed)
  - Male or female and ≥18 years old
  - Histologically confirmed, advanced (unresectable and/or metastatic) and well‑differentiated neuroendocrine tumours (NET) of gastroenteropancreatic (GEP) or presumed GEP origin
  - At least one measurable, somatostatin receptor-positive lesion according to RECIST 1.1 determined by multiphasic computed tomography (CT) or magnetic resonance imaging (MRI; performed within 28 days before randomisation)
  - Somatostatin-receptor imaging must be performed within 12 months before randomisation. Somatostatin receptor-positive lesions are defined as lesions with a visual assessment of uptake greater than the liver
  - If performed, fluorodeoxyglucose positron emission tomography (FDG-PET) CT results for patients with well-differentiated Grade 3 NET must show that FDG avid areas of disease also are avid on somatostatin-receptor imaging
  - Eastern Cooperative Oncology Group performance status of 0–2
  - Having the following laboratory values:
  - Absolute neutrophil count ≥1.5×10^9^/L
  - Platelets ≥75×10^9^/L
  - Haemoglobin ≥9 g/dL
  - Total bilirubin ≤1.5×upper limit of normal (ULN)
    - Patients with a previous diagnosis of Gilbert’s syndrome may be included if the disease is not accompanied by other hepatobiliary disorders, and if the total bilirubin is <3 mg/dL (<51.3 μmol/L) and the direct bilirubin is ≤ULN
  - Aspartate aminotransferase ≤3.0×ULN (if hepatic metastases: ≤5.0×ULN)
  - Alanine aminotransferase ≤3.0×ULN (if hepatic metastases: ≤5.0×ULN)
  - Creatinine clearance ≥40 mL/min defined by the Cockcroft-Gault equation
  - Female patients with childbearing potential must agree to use a suitable method of contraception from screening to the Safety Follow-up Visit

**Exclusion criteria** – Patients meeting any of the following criteria will not be eligible to participate in the clinical trial:

- - Poorly differentiated neuroendocrine carcinoma (NEC), adenocarcinoid, goblet cell carcinoid, large cell NEC, small cell carcinoma, or mixed tumour
  - Previously diagnosed with multiple endocrine neoplasia Type 1
  - Tumour with primary origin outside the GEP tract
  - Documented evidence of disease progression while on treatment (including somatostatin receptor ligand [SRL]) for locally advanced unresectable or metastatic disease
  - Known central nervous system metastases
  - Consecutive treatment with long-acting SRL for >6 months before randomisation
  - Carcinoid symptoms that are refractory to treatment (according to the Investigator’s judgement) with conventional doses of octreotide long-acting release (LAR) or lanreotide autogel (ATG) and/or to treatment with daily doses of ≤600 µg of octreotide immediate release (IR)
  - Previous treatment with >1 cycle (where 1 cycle means ≤28 days on treatment) of targeted therapies such as mammalian target of rapamycin inhibitors (e.g. sirolimus, temsirolimus, or everolimus) or vascular endothelial growth factor inhibitors (e.g. sunitinib, lenvatinib, or cabozantinib), or >1 cycle of chemotherapy or interferon for GEP-NET
  - Treatment of GEP-NET with trans-arterial chemoembolisation or trans-arterial embolisation within 12 months before screening
  - Previously received peptide receptor radionucleotide therapy at any time
  - History of another primary malignancy, except for the following:
  - Stable and well-differentiated microcarcinoma of the thyroid
  - Non-melanoma skin cancer or carcinoma *in situ* of the cervix, uterus or breast, from which the patient has been disease-free for ≥3 years
  - Fully resected, non-metastatic melanoma
  - A primary malignancy that has been completely resected and is in complete remission for ≥5 years
  - Major surgery/surgical therapy for any cause within 1 month before screening, surgical therapy of loco-regional metastases within 3 months before screening, or minor surgery within 14 days before screening. The patient must have recovered from the treatment and be in good clinical condition
  - Hepatic/pancreatic-related exclusion criteria:
  - Active hepatitis. Patients with no significant viral load, no acute signs of inflammation, and no clinical necessity for therapy are allowed, at the Investigator’s discretion
  - Symptomatic cholelithiasis
  - Clinically active or chronic liver disease, including liver cirrhosis of Child-Pugh class B or C
  - Patients with poorly controlled diabetes, as evidenced by haemoglobin A1c >8.0%
  - Cardiac history or current diagnosis of cardiac disease indicating a significant risk of safety for patients participating in the trial, such as uncontrolled or significant cardiac disease, including any of the following:
  - History of myocardial infarction, unstable angina pectoris, or coronary artery bypass graft within 6 months before screening
  - Uncontrolled congestive heart failure
  - Clinically significant cardiac arrhythmias (e.g. ventricular tachycardia), complete left bundle branch block, or high-grade atrioventricular block (e.g. bifascicular block, Mobitz type II, and third-degree atrioventricular block)
  - Long QT syndrome, family history of idiopathic sudden death or congenital long QT syndrome, or any of the following:
  - Risk factors for Torsades de Pointes, including uncorrected hypokalaemia or hypomagnesaemia, history of cardiac failure, or history of clinically significant/symptomatic bradycardia
  - Treatment with concomitant medication(s) with a “known risk of Torsades de Pointes” per www.crediblemeds.org that cannot be discontinued or replaced with safe alternative medication at least 7 days or 5 half-lives (whichever is longer) before the start of trial treatment
  - Patients with a QTc interval corrected by Fridericia’s formula >450 msec for males and >470 msec for females at screening
  - Presence of active or suspected acute or chronic uncontrolled infection, including active human immunodeficiency virus infection
  - Pregnant, lactating, or planning to become pregnant during the trial
  - Any known allergy, hypersensitivity, or intolerance to octreotide, lanreotide, or any related drug, or history of any drug hypersensitivity or intolerance that, in the Investigator’s opinion, would compromise the safety of the patient
  - Clinically significant laboratory abnormalities at screening, which, in the Investigator’s opinion, may prevent the patient from safely participating in the trial
  - Any other contraindicated serious medical condition that, in the Investigator’s opinion, may prevent the patient from safely participating in the trial
  - Any other current or prior medical condition that may interfere with the conduct of the trial or the evaluation of its results in the opinion of the Investigator or the Sponsor’s Medical Monitor
  - Unwilling or unable to comply with the requirements of the protocol or in a situation or condition that, in the Investigator’s opinion, may interfere with participation in the trial
  - Participation in any other clinical trial to test an investigational drug or device within the three months before screening
  - On the staff, affiliated with, or a family member of the personnel directly involved with this trial

**Open-label extension (OLE) inclusion criteria** – Patients must meet the following inclusion criteria (in addition to those detailed for trial participation) to receive CAM2029 (20 mg every week) in the OLE treatment period:

- - Blinded Independent Review Committee (BIRC) confirmed disease progression
  - Voluntary and valid written informed consent to participate in the OLE treatment period (before any procedures are performed)
  - At least 6 months of treatment with either CAM2029 or the comparator in the open-label randomised (OLR) treatment period before documented disease progression
  - Female patients of childbearing potential must (continue to) be willing to use an acceptable method of contraception until the Safety Follow-up Visit

**OLE exclusion criteria** – Patients meeting the following exclusion criteria (in addition to those detailed for trial participation) will not be eligible to participate in the OLE treatment period:

- - Unresolved, drug-related serious adverse event that contraindicates treatment with CAM2029 (according to the Investigator)
  - Clinically significant symptoms, medical conditions, rapid clinical deterioration, or other circumstances that would preclude compliance with the protocol, adequate cooperation in the trial, or may prevent the patient from safely participating in the trial (according to the Investigator)
  - Pregnancy

Abbreviations

| **Abbreviation** | **Definition** |
| --- | --- |
| AE | Adverse event |
| ATG | Autogel |
| BIRC | Blinded Independent Review Committee |
| CR | Complete response |
| CT | Computed tomography |
| DCR | Disease control rate |
| ECG | Electrocardiogram |
| ECOG | Eastern Cooperative Oncology Group |
| EORTC QLQ-C30 | European Organization for Research and Treatment of Cancer’s Core Quality of Life Questionnaire |
| FDG | Fluorodeoxyglucose |
| GEP-NET | Gastroenteropancreatic neuroendocrine tumours |
| HCV | Hepatitis C virus |
| HIV | Human immunodeficiency virus |
| LAR | Long-acting release |
| NEC | Neuroendocrine carcinoma |
| OLE | Open-label extension |
| OLR | Open-label randomised |
| ORR | Overall response rate |
| OS | Overall survival |
| PCR | Polymerase chain reaction |
| PET | Positron emission tomography |
| PFS | Progression-free survival |
| PFS2 | Progression-free survival 2 |
| PFS-ext | Progression-free survival in the Extension Treatment Period |
| PGI-S | Patient Global Impression of Severity |
| PK | Pharmacokinetics |
| PR | Partial response |
| PROs | Patient-reported outcomes |
| QLQ-GINET21 | Quality of Life Questionnaire – Neuroendocrine Carcinoid Module |
| QoL | Quality of life |
| QW | Every week |
| RECIST 1.1 | Response Evaluation Criteria in Solid Tumours version 1.1 |
| SD | Stable disease |
| SF-36 | Short Form-36 Survey |
| SRL | Somatostatin receptor ligand |
| TSQM | Treatment Satisfaction Questionnaire for Medication |
| ULN | Upper limit of normal |
